# Simplify p53: just an activator

**DOI:** 10.18632/oncotarget.3032

**Published:** 2014-12-16

**Authors:** Kurt Engeland

**Affiliations:** Molecular Oncology, Medical School, University of Leipzig, Leipzig, Germany

‘Simplify your life.’ In research - contrasting such a popular concept - we are challenged by an ever-expanding plethora of observations and accumulation of knowledge. This leads to the development of increasingly complex models trying to explain all aspects of our world that are subject to scientific endeavor. Recently, a well-known object of study from the life sciences, the tumor suppressor p53, has emerged as an example for which diversity of its apparent functions has actually decreased.

p53 has long been described to function as a transcription factor. Initially, the cyclin-dependent kinase inhibitor gene *CDKN1A* (p21^WAF1/CIP1^) had been discovered as transcriptionally activated by p53 [1]. Similar to this early observation, many genes were detected to be activated by the tumor suppressor. Often the genes upregulated by p53 serve in blocking the cell cycle or participate in apoptosis induction. In the years following the discovery that p53 acts as a transcriptional activator, it became apparent that p53 is also able to downregulate gene expression [2]. Interestingly, genes repressed by p53 are often involved in control of the cell cycle as well.

Following the observation that p53 can serve as an activating as well as a repressing transcription factor, the question arose how a transcription factor could serve as an activator for one group of genes and as a repressor for another. A diverse spectrum of mechanisms was postulated to encompass these opposing functions [3]. Generally, two principal mechanisms were suggested. The first mechanism assumed direct p53 binding to the target gene. Direct binding is also the central part of the mechanism generally accepted when p53 serves as a transcriptional activator [1;3;4]. Proposed mechanisms of repression through direct p53 binding to the target gene include replacement of activating transcription factors, interference with transcriptional activators, utilization of variations from the canonical p53 binding site or recruitment of repressing factors through p53 [3]. The second class of repression mechanisms does not involve direct binding of p53 to its target genes. This mechanistic group includes complex formation of p53 with other transcriptionally active proteins in solution or with the complex-forming partners bound to DNA. Thereby p53 blocks activating transcription factors bound to DNA or keeps them detached from the DNA through complex formation in solution. Another possible mechanism suggests interference of p53 with the basal transcriptional machinery. Furthermore, several indirect mechanisms were described requiring p53-dependent expression of *CDKN1A* (p21^WAF1/CIP1^) or genes of non-coding RNAs [3]. Although some of the proposed mechanisms held contradictions or were not fully experimentally validated, we were left with the impression that p53 exhibits a multitude of talents serving both as a transcriptional activator and repressor. However, technical progress led to the reassessment of p53's potential modes of operation.

Advance in analysis of large genome-wide data sets has revolutionized the way we interpret biological processes. Also for p53, as a prominent transcription factor, genome-wide chromatin immunoprecipitation (ChIP) and mRNA expression data are available. This provides the opportunity to perform bioinformatic analyses of many independent experiments and to question some of the mechanisms discussed for transcriptional regulation by p53. Such a meta-analysis was recently published in *Cell Cycle* [5]. In a computational analysis, results from six reports on p53-dependent RNA expression were correlated with data from six genome-wide p53 binding studies. The meta-analysis led to the main conclusion that p53 does not bind to genes which are repressed by the tumor suppressor.

It serves as a positive control for the meta-analysis that the correlation between the increase in RNA expression and p53 binding to genes which it activates is very strong. It had been noticed in earlier reports that there are contradictions between the downregulation of p53-repressed genes and published observations on p53 binding to such genes when a mechanism with direct p53 binding was suggested [5]. However, the current report addresses these issues, resolves them for most genes and comes to the conclusion that p53-dependent repression is always indirect.

The paper also offers an alternative to direct repression, at least for a large fraction of the downregulated genes. The meta-analysis provides evidence that the p53-p21-Cdk/Cyclin-RB/E2F or p53-p21-Cdk/Cyclin-DREAM pathways [6] link p53 activity to the downregulation of target genes [5]. The first pathway results in the established binding of RB/E2F complexes on E2F sites in the promoter. However, the novel p53-p21-Cdk/Cyclin-DREAM pathway leads to repression through the only recently described binding of the MuvB core of the DREAM complex to CHR elements in the target promoters [6;7]. The p53-p21-Cdk/Cyclin-DREAM pathway had recently been established with the *Cyclin B2* gene as an example [6].

The report by *Fischer et al*. simplifies the model by which p53 operates as a transcription factor – p53 is simply an activator.
